# High Expression of RAR*β* Is a Favorable Factor in Colorectal Cancer

**DOI:** 10.1155/2019/7138754

**Published:** 2019-03-03

**Authors:** Wei Wang, Shuang Liu, Chunyi Jiang, Yan Wang, Huijun Zhu, XuDong Wang

**Affiliations:** ^1^Department of Pathology, Affiliated Hospital of Nantong University, Nantong, 226001 Jiangsu, China; ^2^School of Medicine, Nantong University, Nantong, 226001 Jiangsu, China; ^3^Department of Clinical Biobank, Affiliated Hospital of Nantong University, Nantong, 226001 Jiangsu, China; ^4^Department of Laboratory Medicine, Affiliated Hospital of Nantong University, Nantong, 226001 Jiangsu, China

## Abstract

RAR*β* plays a critical role in cancer progression and is associated with several types of human cancer. It remains unclear, however, whether it is linked to the clinicopathological parameters of colorectal cancer (CRC). We therefore determined the expression of RAR*β* protein in patients with primary CRC and examined its relationship with clinical outcomes. RAR*β* expression in 234 samples of CRC patients and matched benign noncancerous tumors was detected by immunohistochemistry. RAR*β* mRNA expression was confirmed using the TCGA and Oncomine databases. COX regression analysis and Kaplan–Meier survival analysis were performed to determine the relationship between RAR*β* expression and CRC prognosis. Our results show that high expression of RAR*β* correlated with better prognosis in CRC patients. RAR*β* expression in CRC specimens was clearly lower than in peritumoral specimens (30.8% vs 58.8%, *p* < 0.001) and significantly correlated with gender (*χ*^2^ = 3.926, *p* = 0.048), tumor differentiation (*χ*^2^ = 5.978, *p* = 0.014), and tumor stage (*χ*^2^ = 6.642, *p* = 0.036). Multivariate analyses further revealed that low RAR*β* expression (*p* = 0.001), distant metastasis (*p* = 0.001), tissue differentiation (*p* = 0.006), and tumor stage (*p* = 0.002) were associated with overall survival in CRC patients. In addition, Kaplan–Meier analysis indicated that increased RAR*β* expression in cytoplasm (*p* = 0.001) and early tumor TNM stage (*p* = 0.030) was associated with a more favorable outcome in patients with CRC. In conclusion, RAR*β* expression was strongly correlated with several clinicopathological factors of CRC and may represent a favorable prognostic marker in patients with CRC.

## 1. Introduction

Colorectal cancer (CRC) is the third most prevalent cancer globally after lung and breast cancer [1, 2], and its incidence has increased in recent years along with an increasing number of old people [3]. According to the literature, 45.5% of the incidence and mortality of CRC in China is attributable to unhealthy lifestyles and environmental risk factors including obesity, alcohol consumption, smoking, physical inactivity, and dietary factors [4]. The treatment and prognosis of approximately half of CRC patients with late-stage disease are unsatisfactory owing to resistance to treatment and distant metastasis [2]. Among CRC patients with liver metastases, less than 20% are suitable candidates for surgical intervention [5]. The inhibition of apoptosis in the epithelium of the CRC may contribute to malignancy [6], although the precise molecular mechanisms responsible for metastasis in CRC remain unclear [7]. The identification of novel diagnostic markers and risk factors for CRC is therefore required.

Retinoic acid (RA), a metabolite or analog of vitamin A, is the active form of retinoid with biological functions in processes including cellular proliferation and differentiation, exerted through its receptors, A receptor (RAR) and X receptor (RXR). These receptors each include an *α*, *β*, and *γ* component. Several studies have indicated that RA might be effective in the treatment of malignancies. All-trans RA-based chemotherapy has been used as standard therapy for promyelocytic leukemia, with remission rates of more than 90% [8, 9]. Previous researchers have also reported that RAR*β* protein is epigenetically silenced with tumor progression, indicating that RAR*β* might be a tumor-suppressor gene [10–12].

Some studies have shown that decreased or absent RAR*β* expression correlates with an increase in metastatic processes in various tumor types, including breast cancer and cancers of the digestive tract. However, little has been reported on its role in CRC, particularly its tumor suppressor function [10–12].

In this study, we evaluated the level of RAR*β* expression in human CRC tissues compared with corresponding tumor-adjacent tissues from the same patients and assessed the relationship between RAR*β* expression and clinical prognosis.

## 2. Materials and Methods

### 2.1. Patients and Tissue Microarray Analysis

Formalin-fixed and paraffin-embedded tumor samples and corresponding normal adjacent tissues were obtained from CRC patients treated at the Affiliated Hospital of Nantong University between January 2008 and December 2008. The included patients did not receive neoadjuvant radiation, neoadjuvant chemotherapy, or immunotherapy. The work described has been carried out in accordance with The Code of Ethics of the World Medical Association (Declaration of Helsinki), and the study protocol was approved by the ethics committee of the *Affiliated Hospital of Nantong University.*

Immunohistochemistry (IHC) with tissue microarrays (TMAs) was used to assess RAR*β* expression in 234 patients with CRC. The average patient age was 60 years, (range: 34–92 years). Clinicopathological parameters including sex, age, tumor stage, tumor diameter, lymph node metastasis, and differentiation were obtained from patient medical records. Overall survival was evaluated from the date of surgical resection until death or the end of the follow-up period. Tumor stage was evaluated according to CRC TNM staging guidelines, version 7 of the World Health Organization classification.

TMAs were constructed as described previously [13]. Following hematoxylin and eosin staining, representative tumor areas were labeled in paraffin blocks. Specimen samples (2 mm in diameter) were prepared and sequentially aligned into fixed paraffin blocks, from which 4 *μ*m optimum sections were prepared [13].

### 2.2. Immunohistochemical Analysis

We performed IHC on CRC tissues using a primary antibody against RAR*β*. In brief, tissue sections were deparaffinized and antigen retrieval was performed by boiling under high pressure in 0.01 M citrate buffer (pH 7.0) for 5 minutes. Next, sections were incubated with goat serum reagent in phosphate buffered saline (PBS) for 10 minutes to block nonspecific binding. Tissues were then incubated with primary rabbit anti-RAR*β* antibody (1 : 300, ab124701; Abcam, Cambridge, MA, this rabbit monoclonal antibody is against human RAR*β* amino acids 400 to the C-terminus following the instruction of the manufacturer) overnight at 4°C and subsequently incubated with goat anti-rabbit HRP (Dako, Carpinteria, CA) as secondary antibody for 30 minutes, followed by three washes with PBS. Immunostained CRC sections were evaluated by two experienced pathologists under blinded conditions.

### 2.3. Statistical Methods

The percentage of RAR*β*-positive cells was scored from 0%–100%. The staining intensity of RAR*β*-positive cells was defined as follows: 0, 1, 2, or 3 for negative, weak, moderate, or strong intensity, respectively. To evaluate statistical significance, an appropriate cut point was established based on RAR*β* expression score and overall survival using the X-tile software program (the Rimm Lab, Yale University, New Haven, CT). The final sum of percentage of RAR*β*-positive cells and intensity score represented the RAR*β* immunostaining score.

A chi-square test was used to compare protein expression of RAR*β* in CRC tissues with corresponding tumor-adjacent tissues and to evaluate the association between RAR*β* protein expression and clinicopathological variables. Kaplan–Meier survival curves and the log-rank test were used to calculate the overall survival rate. The prognostic significance of univariate data models was examined using a multivariate Cox regression analysis. We did univariate analysis using clinical parameters like gender, age, RAR*β* expression, and others, respectively. Then, all clinical parameters that meet *p*value < 0.05 in univariate analysis were recruited into multivariate analysis. *p*value < 0.05 was accepted as statistically significant. All analyses were performed using IBM SPSS software version 22 (SPSS Inc., Chicago, IL).

### 2.4. Validation of RAR*β* Expression in CRC

To validate RAR*β* expression and its relationship with clinical parameters in CRC, we explored expression of RAR*β* mRNA in online database Oncomine (https://www.oncomine.org). Two CRC datasets were used (Bittner Colon dataset: 373 samples and TCGA dataset: 237 samples).

## 3. Results

### 3.1. RAR*β* Protein Expression in CRC Tissues

**Figure 1:**
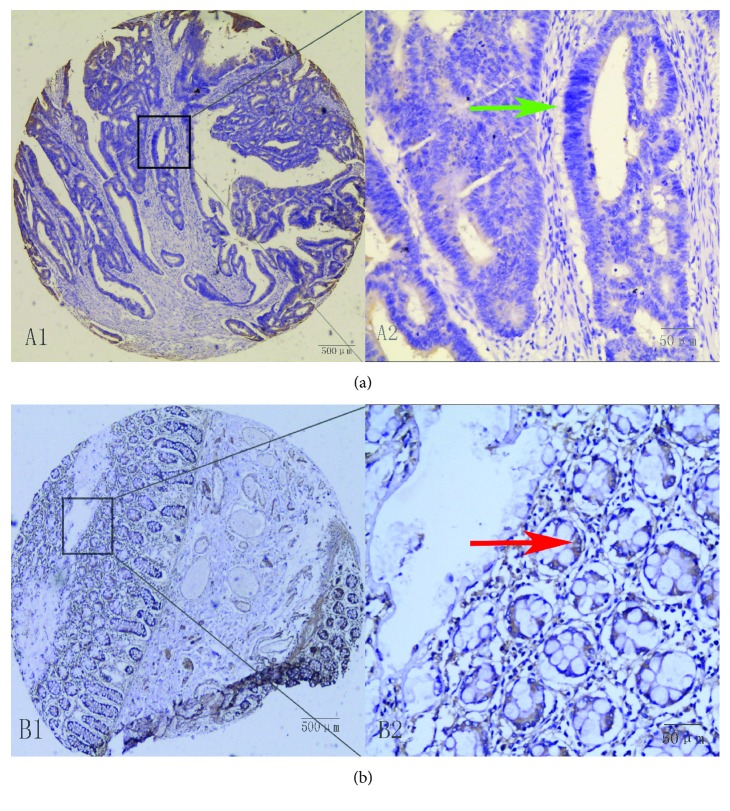
Immunohistochemical staining was carried out on paraffin-embedded 4 *μ*m sections, and representative patterns of RAR*β* protein expression in colorectal cancer (a) and peritumoral tissue (b) are shown. In colorectal cancer tissues, no expression was observed (A1 and A2). In adjacent normal colorectal tissues, high RAR*β* expression was observed (B1 and B2). A1 and B1: original magnification ×40 (bar = 500 *μ*m): A2 and B2: ×400 (bar = 50 *μ*m).

As shown in Figure 1, RAR*β* expression was primarily observed in the cytoplasm of CRC cells. In CRC samples from 236 patients, RAR*β* expression was detected in 30.8% of samples compared with 58.8% of corresponding peritumoral tissue samples. RAR*β* protein expression was significantly lower in CRC samples compared with adjacent matched tumor tissues (*χ*^2^ = 18.767, *p* < 0.001) (Table 1).

### 3.2. Relationship between RAR*β* Expression and Clinicopathological Characteristics of CRC Patients

We examined the correlation between clinicopathological parameters and RAR*β* immunoreactivity (Table 1). Our data indicate that RAR*β* immunoreactivity was significantly correlated with gender (*p* = 0.048), tumor differentiation (*p* = 0.014), and tumor TNM stage (*p* = 0.036). However, no association was observed between RAR*β* expression and clinicopathological parameters including age, tumor diameter, lymph node metastasis, and distant metastasis (Table 1).

### 3.3. Univariate and Multivariate Analysis of Prognostic Factors

Univariate Cox regression tests revealed that low RAR*β* protein expression (*p* = 0.001), tumor TNM stage (*p* = 0.002), distant metastasis (*p* = 0.001), and differentiation (*p* = 0.006) were inferior prognostic factors for overall survival (Table 2). A multivariate Cox proportional hazards regression model demonstrated that negative or low RAR*β* expression (*p* = 0.001) and TNM stage (*p* = 0.030) were strong predictors of overall survival (Table 2).

**Figure 2:**
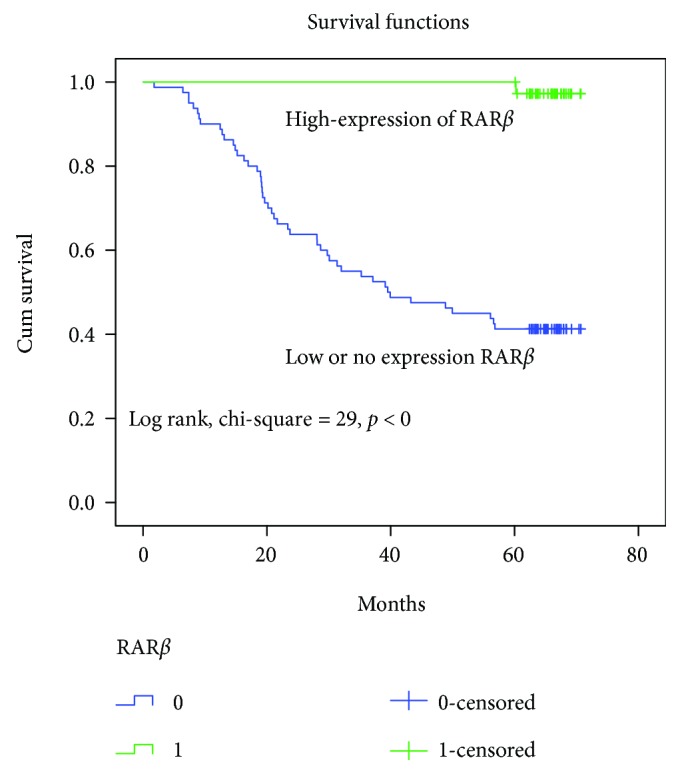
Patients with low or no RAR*β* expression (blue line) exhibited significantly poorer survival compared with the high-expression group (green line).

**Figure 3:**
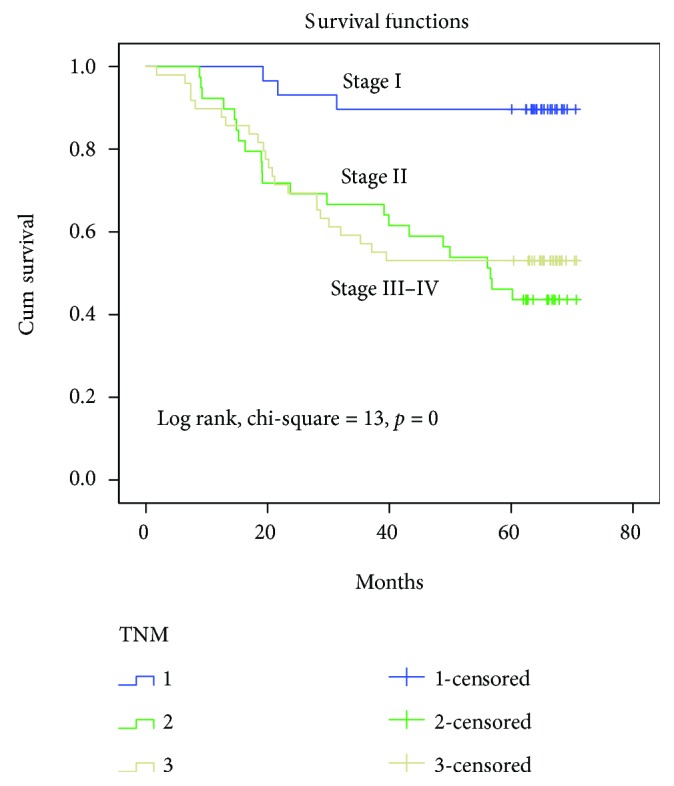
Kaplan–Meier survival curves demonstrate that survival of patients with stage II and stage III–IV CRC tumors is significantly reduced with patients with stage I disease.

Following surgical resection for CRC, Kaplan–Meier survival curves showed that RAR*β*-positive tumors and early-stage cancer (TNM stage I) were associated with longer overall survival compared with RAR*β*-negative tumors and advanced cancer (TNM stages II–IV) in CRC patients (Figures 2 and 3).

### 3.4. Relationship between RAR*β* Protein Expression and Prognosis

**Figure 4:**
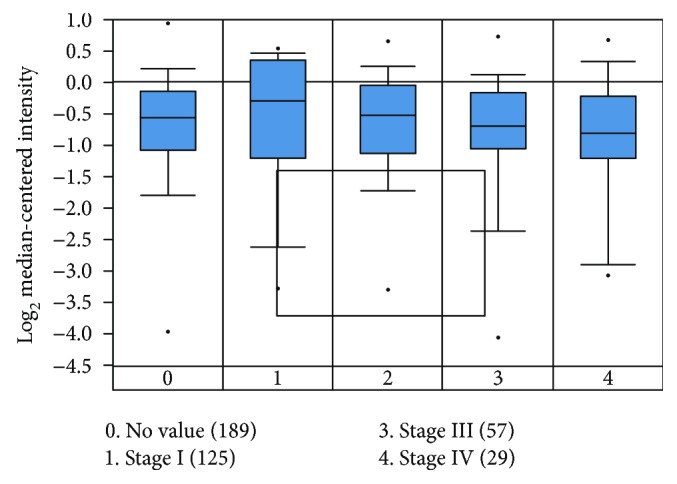
The expression of RAR*β* mRNA correlates negatively with the TNM stages (*p* < 0.05). Among 373 samples of Bittner Colon dataset, we utilized 300 samples of the colon adenocarcinoma which is the most common malignancy of the gastrointestinal tract.

**Figure 5:**
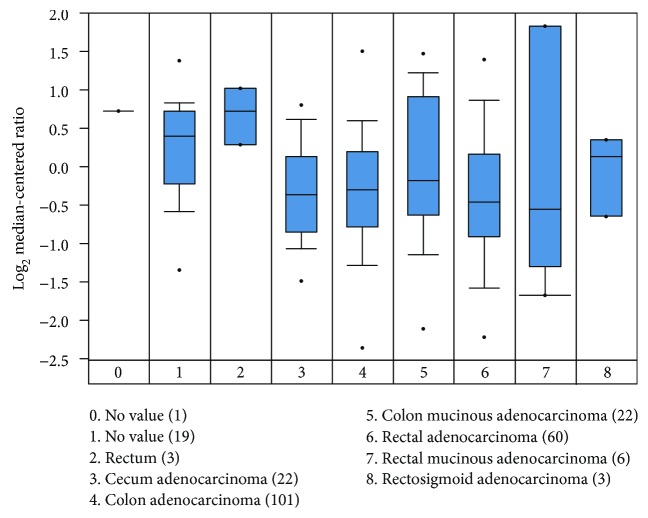
RAR*β* showed higher expression in benign noncancerous tissues compared with colorectal cancer in 237 samples of TCGA dataset.

To assess the correlation between RAR*β* mRNA expression and its clinical parameters of CRC patients, we analyzed data in the Oncomine database and found that the expression of RAR*β* mRNA correlates negatively with CRC TNM stages (*p* < 0.05) (Figure 4). We also analyzed data in the TCGA database and found that low or no RAR*β* expression was shown in CRC tissues compared with high RAR*β* expression in benign noncancerous tissues (Figure 5). These results analyzed using the Oncomine and TCGA databases (Bittner Colon dataset: 373 samples and TCGA dataset: 237 samples) were consistent with our RAR*β* protein expression data in the previous research, which suggested that high RAR*β* expression was a favorable prognostic factor in CRC.

## 4. Discussion

As previously reported [14], RAR*β* is the most commonly expressed RAR subtype in CRC. The expression position of RAR*β* is primarily localized in the CRC cellular cytoplasm. RAR*β* has been shown to function as a tumor suppressor, representing an interesting target in cancer research [10–12]. Indeed, our data suggest that RA-mediated apoptosis in human CRC cells and the significant tumor suppressive effects of RAR*β* may contribute to the chemopreventive actions of retinoid. Recent reports have also indicated that RA, and RAR*β* agonists in particular, inhibits invasion and the migratory potential of breast cancer and endometrial cancer cells [15]. In parallel, the RAR*α*-specific agonist BMS453 significantly reduced the viability of colorectal cells. These results indicate the potential relevance of RAR*β* expression kinetics to the antitumor effect of RAR*β* in CRC. Importantly, the correlation between RAR*β* expression and clinicopathological parameters or prognosis in CRC has been less reported. Given that several studies have shown that marker protein E-cadherin and RAR*β* expression were downregulated in breast cancer samples and that decreased E-cadherin expression is linked to distant metastasis in patients with CRC, we propose that RAR*β* expression may also be relevant in CRC.

An understanding of the multiple prognostic factors impacting survival is critical to the management of cancer after surgical resection [16, 17]. In the present study, we investigated the association between RAR*β* cellular expression and clinicopathological parameters in CRC patients utilizing TMA and appropriate statistical analyses, focusing on the level of RAR*β* expression in CRC specimens compared with adjacent tumor specimens. Our data showed that CRC patients expressing low levels of RAR*β* protein were more likely to have a poor prognosis compared with those expressing high levels of RAR*β* protein in tumors. Although RAR*β* also showed weak expression in tumor-adjacent tissues, high RAR*β* protein expression was associated with a higher overall survival rate, indicating that RAR*β* expression might play a critical role in tumorigenesis among CRC patients.

In consideration of these factors, we demonstrated that high RAR*β* expression correlated with increased overall survival of CRC patients. RAR*β* expression was also associated with gender in CRC patients, an observation that has been insufficiently examined in the previous research. This finding may be associated with the physiological features of patients with advanced CRC. The expression among males is gradually decreasing, but it continues to increase among females, with more proportion. Tumor TNM staging has also been shown to be a significant early prognostic factor in CRC in clinical settings (Table 1). Our findings demonstrated that increased RAR*β* expression and early tumor stage are independent prognostic biomarkers in patients with CRC.

Similarly, previous studies reported a significant correlation between RAR*β* expression and esophageal cancer [18], non-small-cell lung cancer [19, 20], endometrial cancer, and breast cancer [21, 22]. Furthermore, silencing of the RAR*β* gene by promoter hypermethylation is frequently observed in metastatic lung, brain, and bone lesions compared with primary breast cancer [23]. RAR*β* hypermethylation has been found in 92% of endometrial cancers and 75% of endometrial hyperplasias [24]. Previous studies have demonstrated various relationships between clinicopathological parameters and RAR*β* expression in different types of cancer. Taken together, the evidence suggests that RAR*β* expression is reduced in human tumor cells, likely because of the hypermethylation of its promoter [24, 25]. RAR*β* appears to be associated with multiple human regulatory pathways, further indicating its critical role in CRC.

RAR*β* comprises three major isoforms, *β*1, *β*2, and *β*4, with different biological functions. RAR-*β*2 is the most abundant and the major RA-inducible isoform, and thus the term RAR*β* in the literature usually refers to the RAR-*β*2 isoform [26]. Collectively, one of the most important functions of retinoids is their antitumor activity, including the inhibition of tumor growth and the promotion of apoptosis, which has led to their function as chemotherapeutic agents [11, 27]. Although the therapeutic efficacy of retinoids remains controversial based on previous studies, the future study interestingly raises various hypotheses [28].

In summary, previous studies have shown the potential of RA for the treatment of malignancies, given its biological characteristics. Our findings indicate that RAR*β* protein expression is decreased in CRC compared with normal colorectal tissues and is also decreased in tumor tissues compared with corresponding adjacent tissue, indicating that RAR*β* might have a major role in the suppression of tumor invasion and metastasis. Inactivation of the RAR*β* gene might therefore lead to carcinogenesis. It therefore represents a promising candidate for the development of molecular-targeted therapies to ensure a more favorable prognosis for patients with CRC.

As a result of limitations of the present study size and the restriction of the TMA observational findings, further in vivo and in vitro studies are therefore required to examine the anticancer effect of RAR*β* in CRC tumor biology, including the inhibition of cell proliferation, apoptosis, and migration and the significance of RAR*β* and its crosstalk with other markers to increasing the understanding of RAR*β* mechanisms in CRC.

In conclusion, RAR*β* may be utilized as a prognostic factor in the management of CRC and represents a novel therapeutic target in CRC therapy.

## Figures and Tables

**Table 1:** Correlation of RAR*β* expression with clinicopathological characteristics in patients with colorectal cancer.

RAR*β*	*n*	Low or no expression	High expression	Pearson *χ*^2^	*p*
Total	117	81 (69.20)	36 (30.80)		
Gender				3.926	**0.048** ^∗^
Male	74	56 (75.70)	18 (24.30)		
Female	43	25 (58.10)	18 (41.90)		
Age at diagnosis (years)				0.202	0.653
≤60	42	28 (66.70)	14 (33.30)		
>60	75	53 (70.70)	22 (29.30)		
Tumor diameter (cm)				0.396	0.529
≤2	9	7 (77.80)	2 (22.20)		
>2	105	71 (67.60)	34 (32.40)		
	3^a^				
Differentiation				5.978	**0.014** ^∗^
Well	95	61 (64.20)	34 (35.80)		
Poor	22	20 (90.90)	2 (9.10)		
Lymph node metastasis				3.772	0.052
No metastasis	69	43 (62.30)	26 (37.70)		
Metastasis	48	38 (79.20)	10 (20.80)		
Distant metastasis (M)				0.590	0.442
No metastasis	111	76 (68.50)	35 (31.50)		
Metastasis	6	5 (83.30)	1 (16.70)		
Stage grouping with TNM				6.642	**0.036** ^∗^
Stage I	29	15	14		
Stage II	39	27	12		
Stage III-IV	49	39	10		

^a^Immeasurable tumor diameter, 3 cases; ^∗^*p* < 0.05.

**Table 2:** Univariate and multivariate analysis of prognostic factors for overall survival in CRC patients.

Characteristic	Univariate analysis			Multivariate analysis		
	HR	*p* > |Z|	95% CI	HR	*p* > |Z|	95% CI
RAR*β* expression	0.032	**0.001** ^∗^	0.004 0.232	0.038	**0.001** ^∗^	0.005 0.278
Tumor diameter (cm) ≤2 vs >2	4.596	0.132				
Lymph node metastasis No metastasis vs metastasis	1.379	0.268	0.781 2.434			
Distant metastasis (M) No metastasis vs metastasis	5.189	**0.001** ^∗^	2.028 13.272			
Stage grouping with TNM Stage I vs II vs III-IV	1.758	**0.002** ^∗^	1.235 2.503	1.523	**0.030** ^∗^	1.043 2.225
Gender Male vs female	1.584	0.148	0.849 2.953			
Age at diagnosis (years) ≤60 vs >60	1.121	0.708	0.615 2.044			
Differentiation Well vs moderate vs poorly	0.418	**0.006** ^∗^	0.224 0.780	0.687	0.243	0.366 1.289

^∗^ *p* < 0.05.

## Data Availability

This publication is supported by multiple datasets, which are openly available at locations cited in the reference section.
